# Short- and Long-Term Complications of Free Anterolateral Thigh Flap Reconstructions: A Single-Centre Experience of 92 Consecutive Cases

**DOI:** 10.1155/2022/2122956

**Published:** 2022-03-08

**Authors:** Thomas Kidd, Nicholas Platt, Daniel Kidd, Adriaan O. Grobbelaar

**Affiliations:** The Royal Free Hospital, Pond Street, Hampstead, London NW32QG, UK

## Abstract

**Background:**

The anterolateral thigh (ALT) flap has been amongst the most versatile components of the reconstructive surgeon's armamentarium. The authors utilise these flaps for a variety of reconstructive procedures including lower limb reconstruction; postsarcoma excision; and open fractures. Few studies have discussed the extent of recipient site morbidity and subsequent revisional procedures. We will report our experience of the ALT flap in 92 consecutive reconstructions with focus on recipient site complications and revisional procedures.

**Methods:**

Retrospective data collection was done from 92 patients who underwent ALT flap reconstruction—for various large soft tissue defects—at our unit at the Royal Free Hospital, London. We evaluated primary recipient site complications and the requirements for secondary operations after flap transfer.

**Results:**

All flaps survived with the exception of 3 cases (97% survival rate) in which irreversible venous thrombosis was encountered. 16 of 92 patients (17%) required a second recipient site operation for the following: 7 patients experienced major recipient site complications that warranted early return to theatre and 9 patients required a secondary revision thinning procedure(s). 8 of the 16 patients (50%) requiring second operations had construction on their lower leg/ankle/feet (*p* value = 0.10).

**Conclusions:**

Our data demonstrated effective use of the ALT flap in the management of soft tissue reconstructive surgery. Partial flap necrosis was the main complication at the recipient site. In future work, secondary thinning procedures, particularly at the ankle/foot, should be separated from flap-specific complications. Furthermore, we demonstrate tailoring ALT thickness can be performed safely without compromising flap viability.

## 1. Introduction

Large soft tissue defects remain a reconstructive challenge for plastic surgeons. This is, in part, due to the potential composite loss of tendons, muscles, bones, and overlying soft tissues. The affected area is in need of skin coverage with functional reconstruction and filling of 3-dimensional spaces [1, 2]. Since its initial description in 1984, the anterolateral thigh (ALT) flap has been amongst the most versatile components of the reconstructive surgeon's armamentarium [3]. The vascular pedicle is based on either the lateral circumflex femoral artery directly, or the musculocutaneous and septocutaneous perforators of its descending or transverse branch [4, 5]. Its size, long pedicle length, and substantial vessel diameter—as well as its variability in terms of design—renders the ALT flap particularly suitable for large, complex, and challenging soft tissue defects. In addition, a muscle segment can be included on the same pedicle, thus increasing the versatility of the flap and providing additional bulk if required.

Several case series have described its use in the reconstruction of soft tissue defects within the head, neck, and extremities [6–8]. Whilst several reports have discussed the extent of donor site morbidity [9–11], few have explored recipient site complications, which may necessitate revisional operations. These can generally be divided into immediate and late postoperative groups. The former describes those requiring an immediate return to the operating theatre in such cases as microsurgical revision or dehiscence. Late complications would include problems with wound healing (donor or recipient site) and debulking operations, with the latter often being anticipated.

We will report our experience of the versatile free ALT flap in 92 consecutive reconstructions with focus on the recipient site complications and a number of secondary revisional procedures.

## 2. Methods

**Figure 1:**
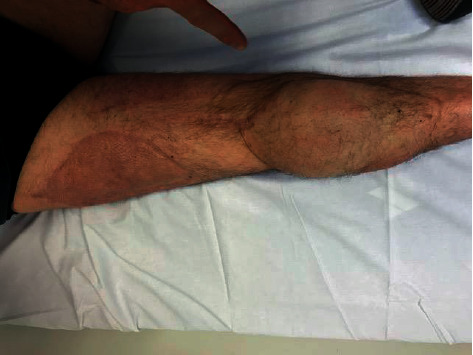
The patient after right leg ipsilateral free ALT flap reconstruction.

The retrospective outcome data from 92 patients who underwent ALT flap reconstruction for various large soft tissue defects were collected at the Royal Free Hospital in London, United Kingdom. This focussed on requirements for secondary operations after flap transfer. ALT free flap dissection was performed as described previously [12, 13]. The perforator vessels are identified using a hand-held Doppler and a defect-sized drawing centred on the perforators. The skin and fascia is incised medially and the suitable perforator(s) identified. We prefer to include at least two perforators as described in the literature [14]. The perforator is traced down intramuscularly towards the main lateral circumflex femoral vessel. Flap elevation is continued suprafascially with a small cuff of fascia included around the perforators. This facilitates wound closure and decreases the incidence of muscle herniation. The complete fascial layer may be elevated in certain instances such as in Achilles tendon reconstruction. The authors employ clinical observation to monitor flaps intraoperatively and do not use techniques described in the literature, such as indocyanine green fluorescence angiography [15]. A postoperative example is shown in Figure 1.

### 2.1. Patients

Between November 2006 and May 2016, 92 patients (53 males, 39 females) with a mean ± SD age of 55 ± 21 years (range 4–91 years) underwent ALT flap reconstruction for various large soft tissue defects (see Table 1). The majority of these involved complex defects secondary to sarcoma reconstruction (42) or trauma and open fractures (25).

## 3. Results

There were 3 cases of flap failure in this group (97 percent flap survival rate), all due to irreversible venous thrombosis. 76 of 92 donor sites (83 percent) were closed directly, and 16 (17 percent) were closed using split-thickness skin grafts taken from either the ipsilateral or contralateral thigh. 7 patients (8 percent) experienced a major complication that required return to theatre within one week of the operation. 9 patients (10 percent) required a return to theatre at a later date for a secondary revision procedure(s). There were no cases of major complications (needing immediate surgery) being caused by the donor site.

### 3.1. Major Recipient Site Complications

As stated previously, 7 patients (8 percent) experienced major recipient site complications necessitating early (<1 week) return to theatre. There were 3 instances of flap failure due to venous thrombosis. The first was on day 1 and resulted in the flap being removed from the fascia and the fascia being left behind as a covering nonvascularized graft. The second occurred at day 2 and required a replacement flap utilising the medial gastrocnemius muscle. The third was at day 4 and required the application of a covering meshed split-thickness skin graft (SSG). There were 2 cases of venous thrombosis where an early return to theatre was needed to ensure the viability of the flaps. One case was a re-look at day 1, but no definitive intervention was needed for minimal venous congestion. The second flap demonstrated small areas of devitalised tissue, and the affected area was debrided and covered with a vacuum-dressing. In another case, there was a small area of devitalised skin at the tip of the graft. The devitalised area was surgically resected, and the remaining flap was advanced and re-sutured. Finally, there was one instance of a retained drain tip that required removal in theatre.

### 3.2. Secondary Revision Procedures

9 patients (10 percent) required a secondary revision procedure. These were all for thinning of a bulky flap for better functional and/or cosmetic outcomes. Methods of debulking included liposuction and surgical thinning.

2 of these patients (2 percent) required a return to theatre for a third time for further debulking. Both of these were located on the foot. One was due to repeated excess bulking and the second due to excess bulk and hyperextension at 5^th^ MTPJ requiring tenolysis.

### 3.3. Secondary Procedures vs Location

8 of the 16 patients (50 percent) requiring second operations had defects on their lower leg/ankle/foot. Analysis using a Chi-squared test of re-operation rates comparing lower leg/ankle/foot vs elsewhere in the body gave a *p* value of 0.10. Of these, 6 patients (38 percent) had secondary revision procedures performed due to excess bulk. Excess tissue can result in difficulties with footwear and/or gait, and such procedures are a common consequence for this recipient site and are described in previous reports [16]. They may not necessarily be considered a “complication” of the procedure.

3 patients (19 percent) requiring second operations had reconstruction on the knee. 1 was due to a retained drain tip, and 2 were for venous thrombosis (1 requiring a re-look and 1 requiring debridement with a vacuum dressing). 2 patients (13 percent) of those requiring second operations had reconstruction on the face. Both were debulking procedures due to excess tissue/asymmetry.

The 3 remaining operations were located as follows: the forearm (debulking); thigh (venous thrombosis and flap failure requiring application of meshed SSG); and the lower abdomen (venous thrombosis with flap ischaemia requiring lifting of the flap from the fascia with the fascia used as a nonvascularized graft).

A summary of recipient site complications and secondary procedures can be found in Table 2. The range of the time between initial flap harvesting and the first surgical intervention was 1 to 851 days (mean 267 days; median 91 days).

## 4. Discussion

Extensive composite loss of soft tissues, secondary to tumour excision or complex traumas, results in large tissue defects and functional disability. In general, these tissue defects are normally too extensive to be closed primarily or with local flaps and require free tissue transfer. The overall aim is to improve function and cosmesis and to protect important underlying structures. This process demands tremendous microsurgical skill and relies on the availability of a large and versatile donor flap. Since its initial description, the ALT flap has consistently demonstrated its suitability for various challenging reconstructive purposes. The good results can be attributed to its long pedicle, reliability, versatility, low donor site morbidity, and large potential surface area [17, 18]. In this series of cases, complex soft tissue defects located in the face, scalp, and upper and lower limbs (including foot and ankle) have been reconstructed using free ALT flaps. In each case, the size of the flaps was adequate for complete reconstruction of one continuous defect with the perforators demonstrating good reliability. Whilst numerous reports have discussed complications arising at the donor site, few have discussed morbidity at the recipient site. A large study by Zhang et al. describes both donor site-related and -unrelated complications after ALT flap transfer and included, amongst others, wound dehiscence, flap necrosis, and haematoma formation [18]. However, this study was limited to reconstructions of the head and neck regions exclusively, thus providing no insight into overall complications relating to ALT flap transfers onto other body parts.

The foot and ankle reconstructions warrant particular discussion in the context of free flap transfers since our cohort demonstrated high revision rates within this group. 50 percent of all postoperative revisions were carried out on the foot or ankle area with the majority for debulking reasons. The *p* value of 0.10 was approaching statistical significance and is likely a type 2 statistical error that would resolve with increased numbers. Similar results are demonstrated in other studies [18]. This is not surprising considering the naturally bony structure of this part of the body. Addition of any soft tissue flap to this region will almost inevitably distort the relatively more delicate shape of the ankle and/or foot which affects the type of footwear these patients can use and, consequently, their gait. On the other hand, however, the foot and ankle have a higher requirement for relatively thicker flaps as these areas are associated with increased pressure from shoes and walking. Finding the ideal balance between aggressive flap thinning and flap viability during primary flap transfer remains a difficult feat and therefore, more often than not, results in secondary debulking procedures. Despite documented success of raising functional and aesthetic thin free ALT flaps in the literature, the authors do not routinely perform this in the trauma setting [19]. It is seen safer and more practical to perform this as a secondary procedure rather than risk the complications during initial reconstruction.

More extensive defects involving the loss of parts of bone, muscle, fascia, and overlying soft tissues secondary to trauma or sarcoma resections are most commonly covered using musculocutaneous flaps such as the gastrocnemius muscle, vastus medialis or lateralis muscle flap, or the reversed biceps femoris flap [20, 21]. Despite their easy availability and associated short operating time, these flaps have been shown to be too bulky which compromises cosmesis and functionality [22]. The ALT fasciocutaneous flap on the other hand has been shown to be thin enough to provide adequate cover to complicated structures such as the knee without unduly compromising function and maintaining adequate cosmesis. Therefore, this study reaffirms the effective implementation of the ALT flap in complex reconstruction procedures. The authors recognise that a limitation of this study is not including any flap complications managed nonoperatively, such as with dressings. Future studies should also aim to analyse patient demographics and comorbidities as additional variables to predict donor and recipient flap outcomes.

9 revisional procedures (56 percent) were sarcoma patients. The authors believe this may indicate a relatively poorer tissue quality after wide local resections of tumours and an inherent tendency to develop blood clots (since cancer is prothrombogenic). Furthermore, it may also reflect tissue compromise secondary to auxiliary interventions such as radiation therapy, an established risk factor for wound breakdown and poor healing [23]. More cases of ALT flap transfers after sarcoma resection need to be analysed in order to establish a particular association between sarcoma patients and ALT flap complications.

## 5. Conclusion and Future Directions

Overall, the majority of revisional procedures involved the debulking of flaps as well as the management of flap necrosis and venous thromboses. Secondary flap thinning is often a predictable consequence, especially in the foot/ankle, and should not be considered a complication of the operative procedure. This differentiation is of use when considering possible future management strategies. Flap-related complications may be overcome by tissue engineering cellular constructs. This can allow precise lab control of dimensions and mechanical strength. Furthermore, an in-built vascular network can both recapitulate the hierarchical organization of natural vasculatures and improve their ability to withstand intraoperative trauma. Already, engineered vascularised flaps are gaining increasing popularity within the scientific arena [24, 25], and the authors propose that it may only be a matter of time before the jump from bench to bedside (or, more accurately, theatre) becomes a feasible reality. Until then, we must strive to further analyse the associated complications of current flap transfers, not least to understand how to best fashion the next generation of tissue-engineered free flaps.

## Figures and Tables

**Table 1:** Aetiologies of soft tissue defects requiring ALT flap reconstruction.

Anatomical location of defect	Defect aetiology	Male patients	Female patients	Total
Face	Sarcoma		1	1
	Parotid tumour (mucoepidermoid)	1		1
	BCC	1	1	2

Scalp	SCC	1		1
	Open wound		1	1

Arm	Sarcoma	5	3	8
	Neuroma		1	1
	Defect/scar	1	1	2

Knee	Pigmented villous synovitis	1		1
	Chronic sinus		1	1
	Scar	1		1
	TKR exposed		1	1
	Defects		1	1
	Sarcoma	9	3	12

Leg	Sarcoma	5	13	18
	Soft tissue defect	3		3
	Chronic wound/ulcer	1	2	3
	Nerve sheath tumour	1		1
	Chronic osteomyelitis	2		2

Achilles tendon	Achilles tendon defect	4	1	5
	Scar	3		3
	Tissue defect	4	1	5

Foot/Ankle	Chronic wound	2	2	4
	Sarcoma	2	3	5
	Defect (e.g., trauma and fracture)	5	3	8
Abdomen	Incisional hernia	1		1

**Table 2:** Aetiologies, complications, and management strategies for free ALT flap transfer.

No.	Defect aetiology	Body part	Perforator type	Donor area closure	Complication	Interval from flap harvesting	Management
1	BCC excision nasolabial fold with partial maxillectomy	Face	Descending branch of circumflex artery	Direct	Excess tissue	1 year 8 months	Debulking
2	Sarcoma	Face		Direct	Asymmetry and excess bulk	11 months	Coleman fat transfer and liposuction/debulking
3	Sarcoma	Forearm		SSG	Excess bulk	2 years 4 months	Liposuction and debulking
4	Infected tibial implant	Lower leg/ankle	Lateral femoral circumflex artery	Direct	Excess bulk	1 year 8 months	Liposuction and debulking
5	Sarcoma	Lower leg/ankle		Direct	Excess bulk	2 years 3 months	Liposuction and debulking
6	Sarcoma	Medial malleolus	Lateral femoral circumflex artery	Direct	Excess bulk	1 year 2 months	Liposuction and debulking
7	Chronic wound	Ankle	Lateral femoral circumflex artery	Direct	Excess bulk and tight fascia in superior and inferior areas	2 years 4 months	Liposuction and debulking, fasciotomy
8	Open fractures	Foot		SSG	Excess bulk	1 year 2 months 3 years 5 months	Liposuction and debulking on both occasions
9	Sarcoma	Foot	Lateral femoral circumflex artery	Direct	Excess bulk excess bulk and hyperextension at 5^th^ MTPJ	8 months 1 year 2 months	Liposuction and debulking on both occasions of tenolysis at second revision
10	Exposed TKR	Knee		Direct	Venous thrombosis and small are of flap necrosis	3 days	Debridement of non-viable tissue and coverage with vacuum dressing
11	Incisional hernia	Lower abdomen		Direct	Venous thrombosis, flap ischaemia	1 day	Flap lifted from fascia and fascia used as a nonvascularized graft
12	Sarcoma	Thigh		Direct	Venous thrombosis and flap failure	4 days	Application of meshed SSG
13	Sarcoma	Knee		Direct	Minimal venous thrombosis	1 day	Re-look at pedicle
14	Sarcoma	Lower leg/ankle		Direct	Venous thrombosis and flap failure	2 days	Medial gastrocnemius muscle flap
15	Burn scar contracture with cellulitis	Ankle	Lateral femoral circumflex artery	Direct	Small area of devitalised skin	1 week	Resection of devitalised skin and re-suture
16	Sarcoma	Knee		Direct	Retained drain tip	4 days	Removal of retained drain tip in theatre

Abbreviations: SSG, split-thickness skin graft; MTPJ, metatarsophalangeal joint; TKR, total knee replacement.

## Data Availability

The retrospective data used to support the findings of this study are available from the corresponding author upon request.

## References

[1] Demirkan F., Chen H.-c., Wei F.-c. (2000). The versatile anterolateral thigh flap: a musculocutaneous flap in disguise in head and neck reconstruction. *British Journal of Plastic Surgery*.

[2] Freedman A. M., Hidalgo D. A. (1990). Full-thickness cheek and lip reconstruction with the radial forearm free flap. *Annals of Plastic Surgery*.

[3] Song Y.-g., Chen G.-z., Song Y.-l. (1984). The free thigh flap: a new free flap concept based on the septocutaneous artery. *British Journal of Plastic Surgery*.

[4] Jeng S.-F., Kuo Y.-R., Wei F.-C., Su C.-Y., Chien C.-Y. (2004). Reconstruction of concomitant lip and cheek through-and-through defects with combined free flap and an advancement flap from the remaining lip. *Plastic and Reconstructive Surgery*.

[5] Kimata Y., Uchiyama K., Sekido M. (1999). Anterolateral thigh flap for abdominal wall reconstruction. *Plastic and Reconstructive Surgery*.

[6] Koshima I., Fukuda H., Yamamoto H., Moriguchi T., Soeda S., Ohta S. (1993). Free anterolateral thigh flaps for reconstruction of head and neck defects. *Plastic and Reconstructive Surgery*.

[7] Kuo Y.-R., Kuo M.-H., Lutz B. S. (2004). One-stage reconstruction of large midline abdominal wall defects using a composite free anterolateral thigh flap with vascularized fascia lata. *Annals of Surgery*.

[8] Kuo Y.-R., Kuo M.-H., Chou W.-C., Liu Y.-T., Lutz B. S., Jeng S.-F. (2003). One-stage reconstruction of soft tissue and Achilles tendon defects using a composite free anterolateral thigh flap with vascularized fascia lata: clinical experience and functional assessment. *Annals of Plastic Surgery*.

[9] Hanasono M. M., Skoracki R. J., Yu P. (2010). A prospective study of donor-site morbidity after anterolateral thigh fasciocutaneous and myocutaneous free flap harvest in 220 patients. *Plastic and Reconstructive Surgery*.

[10] Agostini T., Lazzeri D., Spinelli G. (2013). Anterolateral thigh flap: systematic literature review of specific donor-site complications and their management. *Journal of Cranio-Maxillofacial Surgery*.

[11] Lakhiani C., DeFazio M., Han K., Falola R., Evans K. (2016). Donor-site morbidity following free tissue harvest from the thigh: a systematic review and pooled analysis of complications. *Journal of Reconstructive Microsurgery*.

[12] Marsh D. J., Chana J. S. (2010). Reconstruction of very large defects: a novel application of the double skin paddle anterolateral thigh flap design provides for primary donor-site closure. *Journal of Plastic, Reconstructive & Aesthetic Surgery*.

[13] Marsh D. J., Chana J. S. (2008). Use of the anterolateral thigh flap and combined vascularised fascia lata sling to provide static support following facial nerve resection in head and neck cancer reconstruction. *Journal of Plastic, Reconstructive & Aesthetic Surgery*.

[14] Maruccia M. (2018). Application of extended bi-pedicle anterolateral thigh free flaps for reconstruction of large defects: a case series. *Microsurgery*.

[15] Zhu Y.-L., Wang Y., He X.-Q., Zhu M., Li F.-B., Xu Y.-Q. (2013). Foot and ankle reconstruction: an experience on the use of 14 different flaps in 226 cases. *Microsurgery*.

[16] Koshima I., Nanba Y., Tsutsui T., Takahashi Y., Itoh S. (2002). Perforator flaps in lower extremity reconstruction. *Handchirurgie, Mikrochirurgie, Plastische Chirurgie*.

[17] Maruccia M. (2017). Rectus femoris muscle necrosis: an underrated donor-site complication of free anterolateral thigh flap. *JPRAS*.

[18] Zhang C., Sun J., Zhu H. (2015). Microsurgical free flap reconstructions of the head and neck region: shanghai experience of 34 years and 4640 flaps. *International Journal of Oral and Maxillofacial Surgery*.

[19] Maruccia M. (2017). Suprafascial versus traditional harvesting technique for free antero lateral thigh flap: a case-control study to assess the best functional and aesthetic result in extremity reconstruction. *Microsurgery*.

[20] Siebert C. H., Höfler H.-R., Bruns J., Hansis M. (1996). Reversed Muculus-biceps-femoris-Lappen zur Defektdeckung am distalen Oberschenkel. *Chirurg, Der*.

[21] Pozzobon L. R., Helito C. P., Guimarães T. M., Gobbi R. G., Pécora J. R., Camanho G. L. (2013). Retalhos de rotação para cobertura após artroplastia total de joelho. *Acta Ortopédica Brasileira*.

[22] Cadenelli P., Bordoni D., Radaelli S., Marchesi A. (2015). Proximally based anterolateral-thigh (ALT) flap for knee reconstruction: an advancement propeller perforator flap. *Aesthetic Plastic Surgery*.

[23] Yu S., Zang M., Xu L. (2015). Perforator propeller flap for oncologic reconstruction of soft tissue defects in trunk and extremities. *Annals of Plastic Surgery*.

[24] Shandalov Y., Egozi D., Freiman A., Rosenfeld D., Levenberg S. (2015). A method for constructing vascularized muscle flap. *Methods*.

[25] Kian Kwan Oo K., Chen Ong W., Hui Chi Ang A., Hutmacher D. W., Kim Siang Tan L. (2007). Tissue engineered prefabricated vascularized flaps. *Head & Neck*.

